# Supraclavicular Approach to Ultrasound-Guided Brachiocephalic Vein Cannulation in Children and Neonates

**DOI:** 10.3389/fped.2017.00211

**Published:** 2017-10-05

**Authors:** Zied Merchaoui, Ulrik Lausten-Thomsen, Florence Pierre, Maher Ben Laiba, Nolwenn Le Saché, Pierre Tissieres

**Affiliations:** ^1^Pediatric and Neonatal Intensive Care Unit, Paris South University Hospitals, Le Kremlin-Bicêtre, Assistance Publique Hôpitaux de Paris, Paris, France; ^2^Institute of Integrative Biology of the Cell, CNRS, CEA, University of Paris Sud, Paris Saclay University, Gif-sur-Yvette, France

**Keywords:** central venous catheter, ultrasonography, Doppler, color, children, neonatology, pediatric intensive care

## Abstract

The correct choice of intra vascular access in critically ill neonates should be individualized depending on the type and duration of therapy, gestational and chronological age, weight and/or size, diagnosis, clinical status, and venous system patency. Accordingly, there is an ongoing demand for optimization of catheterization. Recently, the use of ultrasound (US)-guided cannulation of the subclavian vein (SCV) has been described in children and neonates. This article gives an overview of the current use of US for achieving central venous catheter placement in the SCV or the brachiocephalic vein (BCV) in neonates. More than 1,250 catheters have been reported inserted in children and neonates for a cumulated success rate of 98.4% and the complication rate is reported to be low. The technical aspects of various approaches are discussed, and we offer our recommendation of an US-guided technique for SCV and BCV cannulation based on our experience in a large NICU setting. Although the cannulation the SCV or BCV does not substitute the use of peripherally inserted central catheters or umbilical venous central catheters in neonates, it is a feasible route in very small children who are in need of a large caliber central venous access.

## Introduction

Central vascular access is frequently required in critically ill children. It is often an everyday procedure in NICU and PICU units, where percutaneous non-tunneled central catheters are used in urgent, or short-term situations and can remain in place for up to 2 weeks (1). The correct choice of intra vascular access should be individualized depending on the type and duration of therapy, gestational and chronological age, weight and/or size, diagnosis, clinical status, and venous system condition (2).

Peripherally inserted central catheters (PICC) are often used in neonatology in relay of umbilical venous central catheter. However, in acutely ill neonates, a large central venous catheter (CVC) is often warranted. These catheters have a wide range of indications including resuscitation with the need for administration of fluids and vasoactive drugs, the need for prolonged parental nutrition, the need for very frequent blood samples, and when venous access cannot be achieved otherwise. Central venous access (CVA) can be achieved by venipuncture of the internal jugular (IJV), femoral (FV), or subclavian veins (SCV) in newborns. Despite the routine aspect of placing CVCs in NICU and PICU units, the procedure is still associated with complications such as accidental arterial puncture, hematomas, pneumothorax, catheter malposition, and failure of cannulation (3). CVA in smaller children remains technically challenging even in experienced hands (4) and there is an ongoing demand for optimization of catheterization and subsequent management or any immediate catheter-associated complication, including catheter misplacement. The use of bedside ultrasound (US) has been demonstrated to facilitate and secure CVC placement and is now widely used (5). IJV catheterization is perceived as the gold standard, but remains difficult in smaller children weighing <10 kg (6). Therefore, in recent years, an increasing number of data supporting the use of US-guided SCV cannulation in children and neonates has been published (Table 1).

**Table 1:** Summary of overall results form studies published on US-guided SCV cannulations in pediatric populations.

Reference	Patients (*n*)	Weight (kg) range median	Left vs. right sided catheters	Position of the operator	Probe	Results	Complications
Pirotte et al. (7)	23 (25 catheters)	2.2–27.0 (6.1)	67% left sided	Not specified	10 MHz, 2.5 cm “hockey stick” probe	Success rate 100%	No major complications
			33% right sided			First attempt 84%	

Breschan et al. (8)	35 (42 catheters)	0.9–21.0 (6.8)	83.3% left sided	At the child’s head	13–6 MHz, 2.5 cm linear probe	Success rate 100%	No major complications
			16.7% right sided			First attempt 73.8%	

Kulkarni et al. (9)	150	2.7–35.0 (median not reported)	6.5% left sided	At the child’s head	Not described	Success rate 98.77	1.33% (2) arterial puncture 0.67% (1) penumothorax
			93.5% right sided			First attempt not reported	

Breschan et al. (10)	136 (183 catheters)	0.7–10 (3.7)	92.9% left sided	At the child’s head	13–6 MHz, 2.5 cm linear probe	Success rate 98.9%	No major complications
			7.1% right sided			First attempt 82.9%	

Rhondali et al. (4)	37	4.1 (range not reported)	73% left sided	At the child’s head	13–6 MHz, 2.5 cm linear probe	Success rate 100%	No major complications
			27% right sided			First attempt 81%	

Guilbert et al. (11)	42	2–70 (6.5)	Not specified	Not specified	3–11 and a 7–11 MHz	Success rate 97.6%	1 arterial puncture, 1 pneumothorax
						First attempt not reported	

Byon et al. (12)	49	2.6–17.0 (mean 8.1)	Not specified	At the patients right side	8–10 MHz linear “hockey-stick” probe	Success rate 100%	No major complication
						First attempt 93.9%	

Park et al. (13)	11	2.6–12.4 (4.0)	36% left sided	Not specified	25 and 40 mm 6–18 MHz	Success rate 100%	No major complication
			64% right sided				

The aim of the present article is to give an overview of the current use of US for achieving CVC placement in the SCV or the brachiocephalic vein (BCV) in neonates. We will discuss the technical aspects of various approaches and offer our recommendation of an US-guided technique for SCV and BCV cannulation based on our experience.

## US Guidance for Gaining Vascular Access

Ultrasound was first used to facilitate intravascular catheterization almost 40 years ago and for the last 10 years, the development of mobile US machines and improved technology have made bedside US a valuable tool for establishing vascular access, even in routine use (14, 15). The US probe may be placed in either the short-axis view across the diameter or long-axis view along the length of the vessel to allow optimal visualization of the needle entering the vein (1). The benefits of bedside US guidance for percutaneous CVC placement are thought to be due to the real-time visualization of the needle entry in the vein and relationship to surrounding structures. This leads to a reduction of failure rates in both first and total attempts at placement and a complication rates decrease (16–18). The current body of published data from adults supports the use of US over anatomical landmarks for acquiring CVA (19, 20). Similar data seem to suggest the advantage of US for the placement of CVC in children. Several randomized controlled trials comparing the use of US with anatomical landmark placement of a CVC in both neonates and children have reported the superiority of US-guided placement (16, 19, 21–24). Despite that meta-analyses have yet to demonstrate an advantage of US guidance over the anatomical landmark technique in the cannulation of the IJV vein in children (5), the cannulation of the internal jugular vein (IJV) with real-time US guidance is now a standard practice (14, 19). Similarly, the use of US guidance has also been demonstrated to be beneficial in cannulation of the SCV (25), but its neonatal routine use is not yet as widespread in pediatrics (14).

## US Techniques: Long vs. Short Axis View

**Figure 1:**
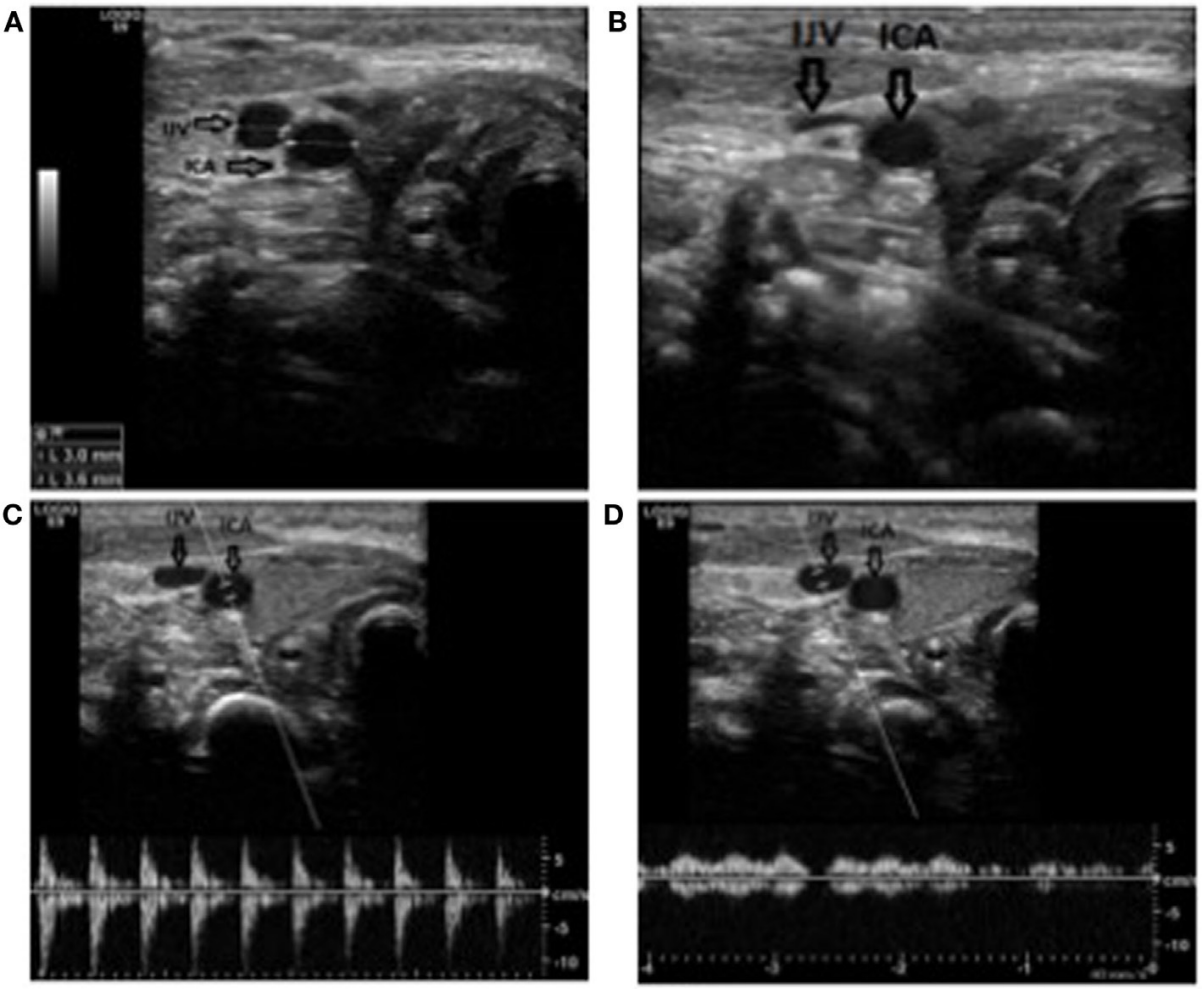
Anatomical identification of the internal jugular vein. **(A)** The vessel in short axis view in a newborn at term. Notice the diameters. IJV: internal jugular vein; ICA: internal carotic artery. **(B)** Notice how the vein collapses under a slight pressure, as oppose to the artery. **(C)** Doppler demonstrating the pulsatile flow in the artery. **(D)** Doppler demonstrating the laminar flow in the vein. Notice how the flow changes with the respiration.

**Figure 2:**
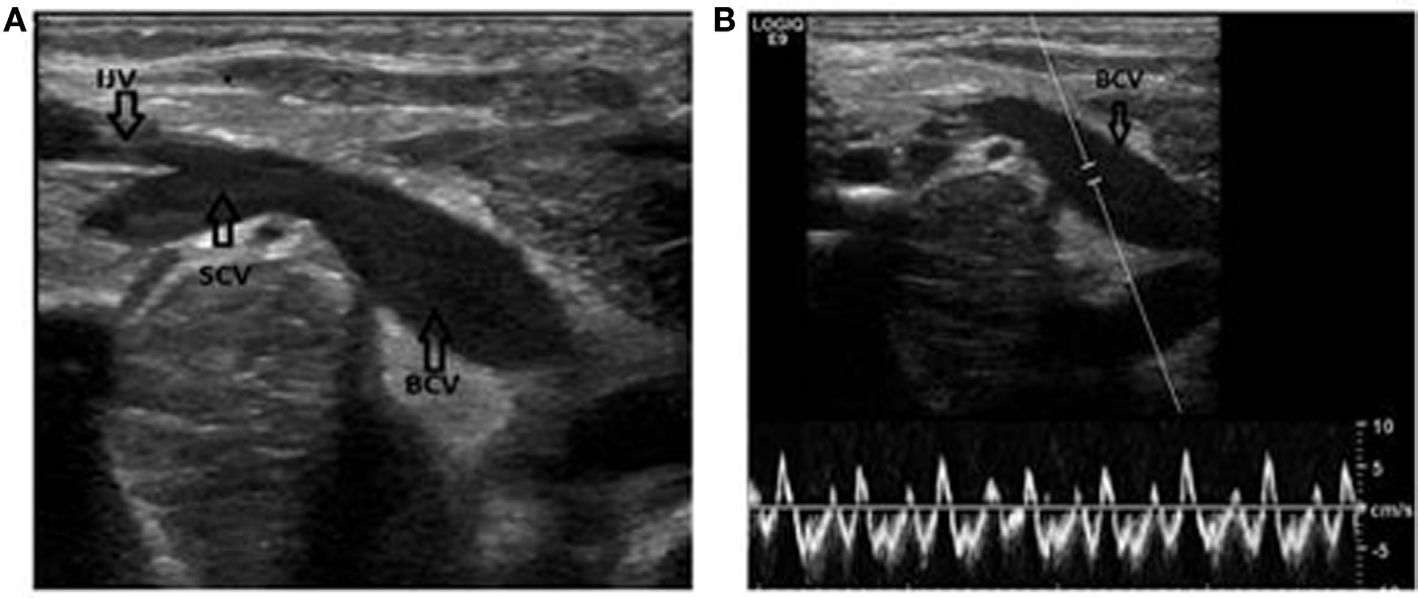
Identification of the subclavian and brachiocephalic veins. **(A)** Long axis view of the veins. Notice the confluence of the brachiocephalic vein and the internal jugular vein. IJV, internal jugular vein; SCV, subclavian vein; BCV, brachiocephalic vein. **(B)** Same patient. The Doppler is used to verify the flow in the vein. Notice how the flow changes with the respiration.

Initial positioning of the US probe perpendicular on the vessel gives rise to a short axis view (Figures 1A–D). The use of this view allows for visualization and identification of the target vessel and the surrounding structures and offers a good midline orientation (15). The “out-of-plane” needle-guided approach that this view offers does not allow for optimal needle tip visualization during the cannulation process. This is due to the fact that the needle reflection (or image) on the screen is also a cross section of the needle, meaning that the needle tip is indistinguishable from any other part of the needle, as they look identical on US (15). However, by placing the US probe parallel to the vessel, the longitudinal, or long axis view, is obtained (Figures 2A,B). This view identifies the target vessel along its length, which allows for the insertion of the needle in an “in-plane” view. This technique gives direct and full visualization of both needle tip and shaft during catheterization. The advantages of this approach are that the needle is easily witnessed entering the vessel, and the guide wire’s direction can be verified, which lessens the risk of mechanical complications and catheter misplacement (15).

## Anatomical Particularities of the SCV and the BCV

**Figure 3:**
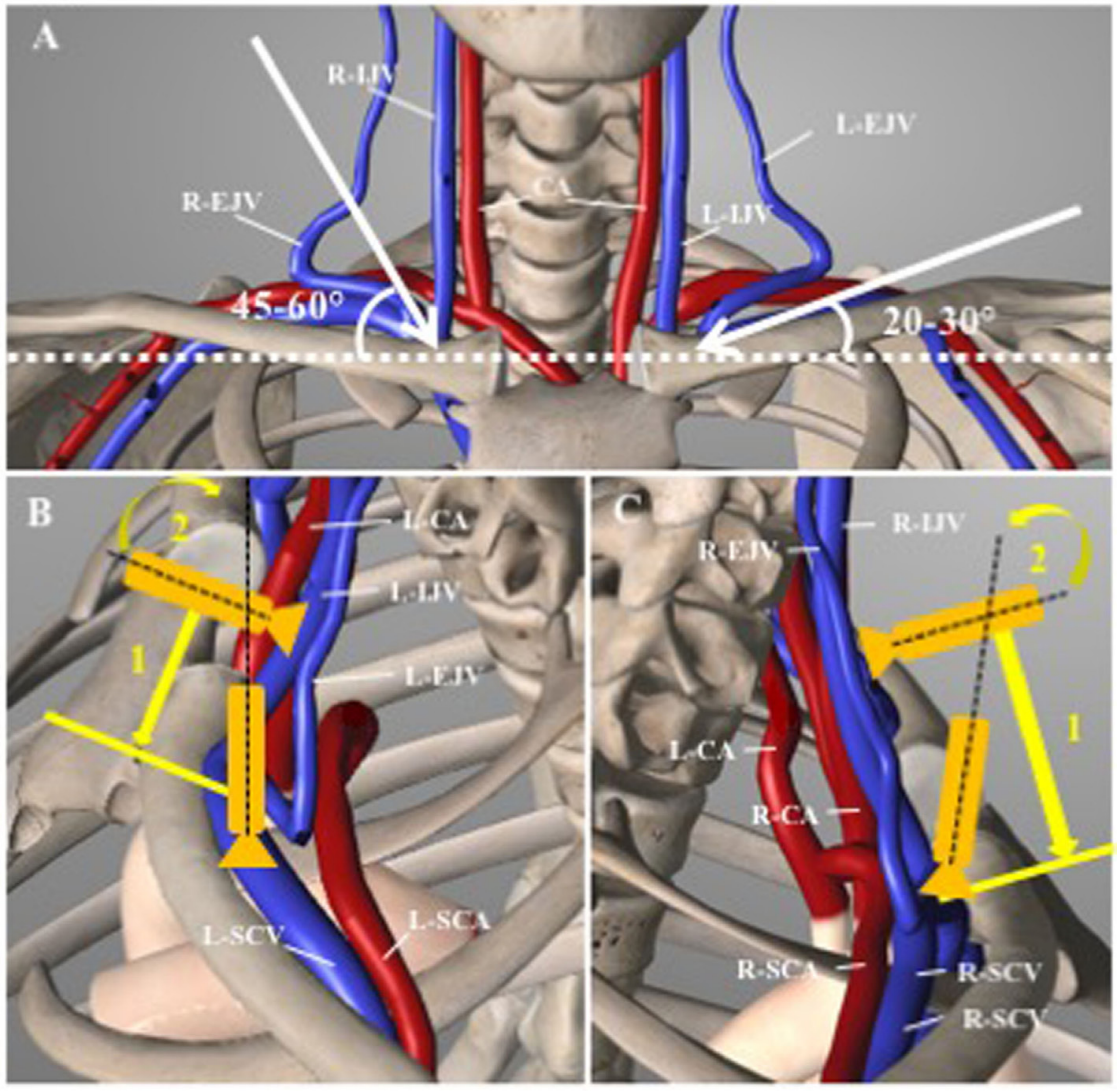
Anatomical view of the cervicothoracic region. **(A)** Frontal view outlining the different angles of puncture between right and left sub-clavian (SCV) and brachiocephalic (BCV) veins. CA, carotid artery; IJV, internal jugular vein; EJV, external jugular vein. **(B)** Left SCV approach: the probe is slided (1) down perpendicular to the IJV and tilted anteriorly (2) toward the L-SCV. Noted that the left subclavian artery (L-SCA) is running posteriorly to the aorta. **(C)** Right SCV approach: Similarly, to the left side approach, the probe is slided down the IJV (1), than tilted anteriorly (2). Noted the close relation of the right-SCV and right-SCA (adapted from Essential Anatomy, V5.0.3, 3D4Medical.com).

The axillary vein courses medially and becomes the SCV at the lateral border of the first rib. It continues its path under the clavicle, arching upwards across the superior surface of the first rib and then inclines medially, downwards, and across the insertion of the anterior scalene muscle; after which, it enters the thorax as it unites with the IJV behind the sternoclavicular joint to become the BCV (25). As with the internal jugular veins, the right and left SCVs are not bilaterally symmetric (Figure 3). The venous course from the left SCV arc through the innominate vein to the superior vena cava (SVC) in a gentle curve, whereas the right SCV makes a more sharply angled turn into the SVC as it is joined by the jugular vein. This sharp angle of the right SCV can lead to difficulties when placing central catheters (26).

## SCV Cannulation in Children

An increasing number of data has demonstrated the feasibility of an US guided supraclavicular approach to the cannulation of the SCV in children and a summary is given in Table 1. More than 1,250 catheters have been reported inserted in children and neonates for a cumulated success rate of 98.4%. Even more important, the complication rates are reported to be low. Furthermore, a fast learning curve (4) has been reported, further adding to the advantages of the technique. Catheters have even been placed in children weighing as little as 710 g (27) and in our experience, US-guided cannulation of the SCV is a safe and often fast method for gaining vascular access, even in neonates.

## Supra vs. Infraclavicular Approach to the SCV

There are limited data comparing supra- to infraclavicular approaches with real-time US guidance (15). One study on adults has demonstrated that identifying the SCV in the supraclavicular region using US is technically easier compared to the infraclavicular region (28). Few studies on supra- vs. infraclavicular approach to the SCV in children has been published, but a study by Byon et al. (12) compared the infraclavicular versus the supraclavicular approach in a cohort of 98 children and found the supraclavicular approach to be associated with a shorter puncture time (36 vs. 48 s), fewer patients needing more than three attempts (6.1 vs. 24.5%), and fewer cases of guide-wire misplacement (0 vs. 20.4%). In our experience, the supraclavicular approach has the advantages of providing sufficient space for placing of the US probe in the supraclavicular fossa, thereby allowing a longitudinal view of the vessels. This allows for a direct real-time visualization of the needle as it advances toward the vein. Finally, when compared to IJV, we find that the supraclavicular approach allows for an easier and better fixation of the catheter, as well as a convenient distance from the mouth, thereby reducing potential contamination.

## Advantages of the SCV and BCV

The SCV diameter usually remains large regardless of hemodynamic and respiratory status, even during hypovolemia (4), making its cannulation easier than other sites in critically ill patients. Furthermore, it offers the advantage of being easier to visualize in smaller children than the IJV in the long axis view for anatomical reasons. This is especially true in smaller neonates, who have short necks and in whom the probe may not “fit” if placed longitudinally (4, 7, 11). Finally, the catheter insertion site exit is conveniently located at a distance from the nasobuccal area, reducing oropharyngeal flora contamination (4, 29). The SCV has been reported to have a reduced risk of infection and thrombosis (30). Also, by aiming for the BCV rather than the SCV, the distance is slightly longer, which allows for real-time adjustment of the needle position as it makes it toward to the vessel. Additionally, the BCV is located further away from the plural dome than the SCV, thereby reducing the risk of accidental pneumothoraxes.

## Right- Versus Left-Sided Catheters as First Choice

Both sides are accessible, and the literature offers no clear data to systematically prefer one to the other. Of the cases summarized in Table 1, a total of 48% was placed in the left and 52% were placed in the right SCV (4, 7–13, 27, 31). The choice of side appears to reflect a personal preference. When placed at the patient side, the left SCV is the more accessible choice, as it allows the operator to hold the US probe in the left hand and the needle in the right hand more freely as stated by Breschan et al. (8, 10). Still, when the operator is placed at the patient head, as Kulkani et al. reported, the right SCV becomes the more logical choice for the same practical reasons (9). Breschan et al. state that the right brachiocephalic vein (BCV) in most cases are shorter the left. Also, the right BCV quickly takes a sharp angled, caudal turn, as opposed to the left, which runs in a significantly more horizontal line (8). The sharper angle and shorter length of the right BCV has been suggested by Breschan et al. to lead to a more difficult US imaging in the longitudinal axis (8). As a consequence, the needle advancement in the in-plane technique is believed to be more difficult on the right side. This may be of greater importance in smaller patient, especially as the left BCV is apparently larger that the right in preterm babies (32). Yet, no clear recommendation as to which side, if any, is first choice can be given based on the current published literature. Still, we suggest the left side as first choice, as we find it to be easier for most right-handed persons. More importantly, the visualization, the more horizontal line of the left BCV is often easier and, in our experience, carries a higher success rate.

## Technique—The Bicêtre Experience

Therefore, we here report our experience and preferred technique of BCV cannulation in children and neonates from a large NICU and PICU in a University Hospital in Paris, France.

### Population

The US-guided supraclavicular approach to subclavian cannulation has been reported successful in children (11), newborns (10), and even low birth weight newborns (27). In our experience, this technique is even applicable in newborns weighing as little as 710 g (27). When done by experienced operators, we find that there are no absolute weight and/or age limit for this technique. In the smaller neonates, we do recommend that the diameter of the SCV is measured and that catheter to vein diameter ratio is less than 0.5.

### Preparation

In high-risk situations, an examination of hemostasis and a thrombocyte count should be done and in case of any bleeding tendency can be anticipated, a correction hereof should be done before the intervention. When urgency dictates immediate cannulation, we recommend cannulation of the IJV or FV, as these sites are easier to compress in case of hematoma. In case of multiple previous vascular catheterization or clinical suspicion of thrombosis, Doppler US of the vessels should be performed.

### Catheters

Standard X-ray opaque, commercially available catheters are recommended. Most often, they are produced in polyurethane, a material that is both flexible and offers a better external to internal diameter ratio than silicone catheters. Polyurethane catheters become even more flexible once warmed and in place. We generally use a 2-Fr (22 G) single lumen, 6 cm catheter (Arrow, Ireland) for children weighing less than 2 kg, a 3Fr catheter (Vygon, France) for children weighing <3 kg and a 3 or 4.5-Fr (Vygon, France) single, double or triple lumen, 6 or 8 cm for children weighing >3 kg.

### US Scanner

The US scanner should be mobile in order for the procedure to be carried out at the bed-side. Furthermore, it should be equipped with Doppler (screening for thrombosis) and zoom function as well as a small (<30 mm) linear high frequency probe (<10 MHz) (33). We use an A L8-18i-D. Intraoperative 8–18 MHz High Frequency Ultrasound Transducer Probe (GE Healthcare, Little Chalfont, UK) connected to a LOGIQ E9 Ultrasound Unit (GE Healthcare, Little Chalfont, UK).

A part from the US scanner, all material should be sterile and disposable. The probe should be covered in sterile US probe sheath. We use a preformed package containing sterile dressing and fields, US probe cover, US gel, scissors, suture thread, and scalpel (Vyset, Vygon, France).

### Sedation

A general anesthesia or profound sedation (with or without assisted ventilation) increases the chances of success and diminishes the risk of complications and breach of sterile barrier that can sometimes be caused by movements of the child. A temporary increase in anesthesia is recommended, if the child is already sedated for other reason. Yet, we find that given the short duration of the procedure a general anesthesia and intubation is often not necessary for the procedure. In case of the latter, we sedate with intravenous ketamine (2–3 mg/kg), possibly intensified by midazolam (10–20 μg/kg) or propofol (1–2 mg/kg) and apply a local analgesia by infiltrating lidocain (0.5–1 mg) In case no intravenous access can be obtained, sedation by rectally administrated ketamine is possible (5 mg/kg, administrated 20–30 before the procedure).

### Position of the Patient and Operator

**Figure 4:**
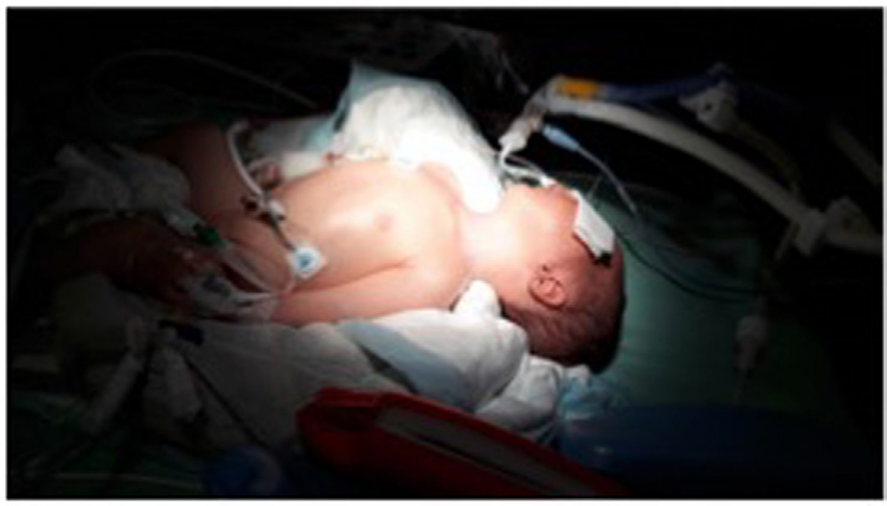
Installation of the patient. Notice the Trendelenburg position, the cushion under the shoulders, and that the head is turned to the right (away from the site of the puncture).

The child is placed on the back in a slightly Trendelenburg position with the arms toward the feet and the head turned 30–45° to the side opposite to the puncture site (Figure 4). A cushion is placed under the shoulders to lift and expose the site of puncture. The operator stands on the same side as the selected puncture site. A position more at the child’s head is preferable for puncture on the right side due to the more descending angle of the vein.

### US Views

Some variations on the technique have been proposed (Table 1). We suggest placing the probe perpendicular on the neck, lateral to the cricothyroid membrane area, to identify the anatomical landmarks, i.e., the internal jugular vein and the carotid artery primarily (Figure 1A). The differentiation between the IJV and the common carotid artery can be made by applying gentle pressure with the US probe and observe how the more compliant vein collapses under the pressure as opposed to the more resistant artery (Figure 1B) and by observing its size variation with respiration. The nature of the vessels can furthermore be confirmed through pulsed Doppler control of vascular flux (Figures 1C,D).

By moving the probe caudally and keeping the vein in view, the confluence of the internal jugular vein and the SCV can be identified along with the clavicle and the pleural line. The probe now rests in the supraclavicular fossa and by slightly tilted the probe postero-anteriorly, one can obtain an ideal view of the SCV in plane (Figure 2A). The SCV can be confirmed as it is in direct contact with the pleura dome (that in turn can be identified by the movements of the two sliding lamina), and is found anteriorly to the artery. The nature of the vein can once again be confirmed by Doppler (Figure 2B).

### Sterile Technique

All published data used some variation of a standard sterile technique, aseptic cleaning of the skin, and surgical cover of the patients. Whereas most reported, the use of applying the coupling gel on the probe and secondarily covering the probe in a sterile sheath, Kulkarni et al. (9) used an open-ended sterilized plastic sheath and covered the probe in sterile Tegaderm once it emerged from the plastic sheath, ensuring that no air bubbles were entrapped. They then used a few drops of saline as coupling gel whereas others used sterile US gel (8). We suggest a standard sterile technique, including aseptic wash of the skin and complete coverage of the patient and bed/incubator using sterile surgical covers leaving a hole on the “operation field.” The US probe should be covered with a sterile sheath and the operator should wear sterile protection as per standard recommendations.

### Puncture Technique

**Figure 5:**
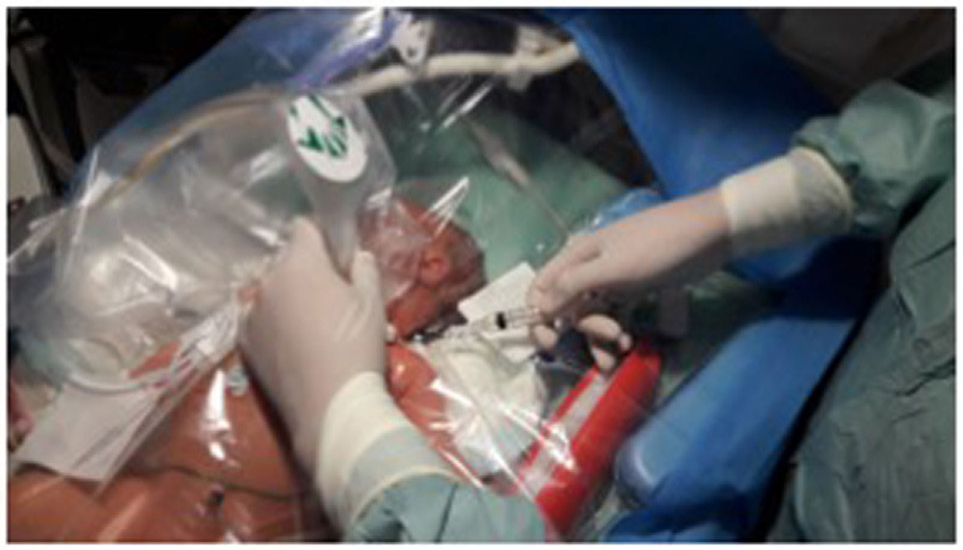
Demonstration of the position for puncture. Notice angle of the needle in relation to the ultrasound probe.

**Figure 6:**
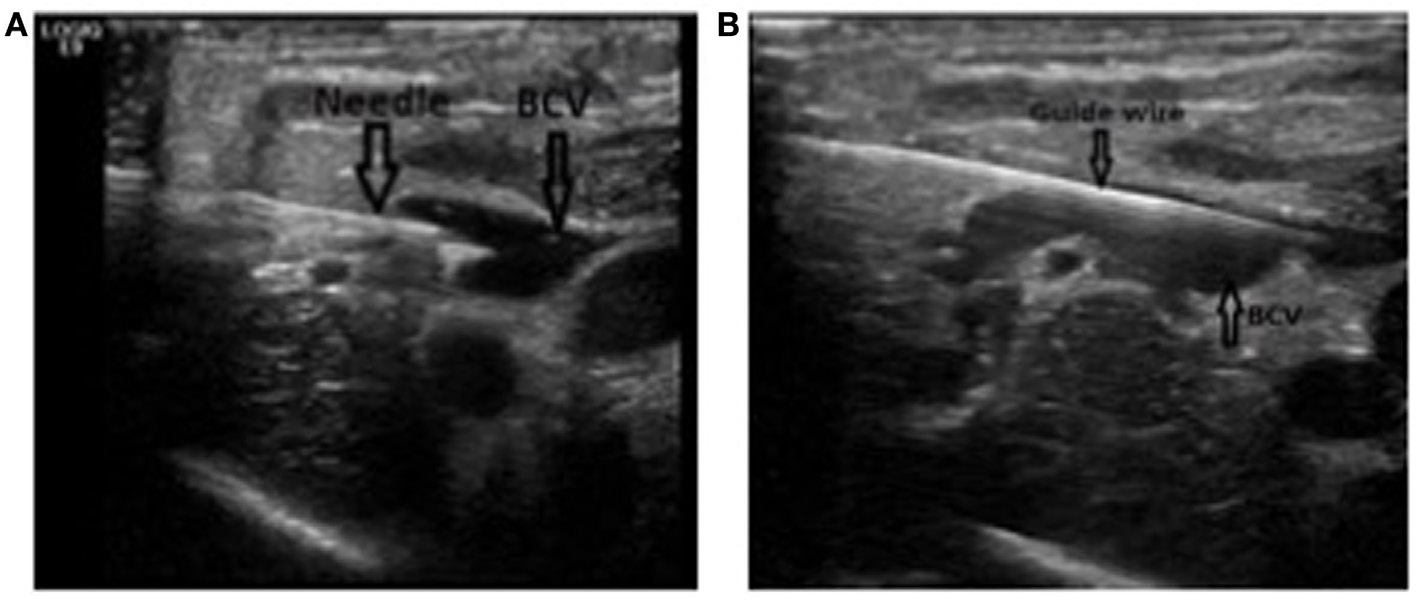
Puncture and catheterization of the brachiocephalic vein. **(A)** Long axis view of the brachiocephalic vein with the needle in place in the vein. Notice how the two borders of the needle can be seen. **(B)** Long axis view of the brachiocephalic vein with the guide wire in place in the vein. The needle has been removed.

A classic *Seldinger* technique is used for puncture. The use of a standard IV catheter (7), or a needle with (11) or without (31) an attached syringe has been described. We suggest the use of a 2-Fr needle loosely attached to a 5-mL syringe with a standard luer lock, as the syringe gives the needle a handle that makes the manipulation of the needle easier and allows for verification of the position through aspiration. The use of the luer lock prevents the needle and the syringe to be too tightly attached during the advancement of the needle, which in turn can make the removal of the syringe more difficult. Furthermore, the venous pressure in neonates often is too low for a spontaneous return of blood once the vessel is punctured. Therefore, the use of a syringe that allows for gentle aspiration during advancement of the needle makes identification of puncture easier. The operator’s dominant hand is used for puncture and the other for positioning the US probe (Figure 5). Point of entry is lateral to the probe and the needle is advanced under constant real-time US observation. Once the intravenous position is verified by US (Figure 6A) and by a return of blood in the syringe, the position of the needle is now carefully held in place by the hand that previously was used to hold the US probe.

### Advancement of the Guide Wire

The syringe is removed by screwing counterclockwise, and a guide wire can be advanced through the needle. The advancement of the guide wire should not be forced and in case of resistance, the angle of advancement should be changed slightly. In case of persistent resistance, extra-vasal position must be suspected and a withdrawal of the guide wire and subsequent re-puncture must be considered. The correct intravenous placement and the trajectory of the guide wire must then be verified by US (Figure 6B). For real-time US-guided identification of the vessels and puncture of the vein, please see Video S1 in Supplementary Material.

### Dilation

Subsequently, after a small incision in the skin (often not necessary in the smaller babies), the subcutaneous tissue and the vein can be dilated using the provided dilators in the catheter package. Failure to do so can lead the difficulties in advancing the catheter over the guide wire due to diameter mismatch. A dilation of the first 2–3 cm is often sufficient.

### Advancement of the Catheter

**Figure 7:**
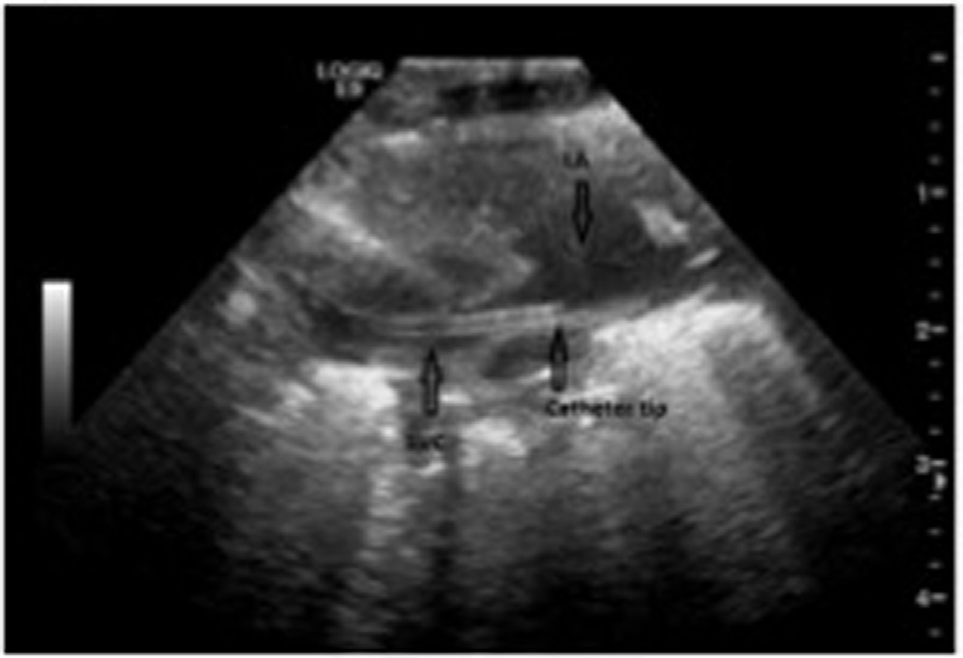
Catheter tip positioning. Transthoracic echocardiographic examination demonstrating the catheter tip in the superior vena cava. SVC, superior vena cava; LA, left atrium.

After removing the dilator, the catheter is introduced over the guide wire to the estimated length. Correct intravenous position can be verified by free in- and out-flow of the catheter. Its position in the SVC and the absence of accidental pleural puncture, i.e., the presence of pneumothorax is subsequently confirmed clinically, by US (Figure 7), and by X-ray examination of all patients.

### Fixation of the Catheter

The catheter must be fixed to the skin by non-reabsorbable sutures and subsequently covered by transparent sterile dressing that allows for observation of the point of puncture. The dressing is not changed but left in place for the duration of the catheter. It is changed if it starts to peel off or is otherwise compromised.

## Particularities of Neonatal Central Vein Cannulation

The small diameter of the vessels in neonates makes central vein cannulation potentially difficult. It is sometimes difficult to distinguish between arteries and veins in the smallest of babies, but with the aid of US guidance, one can apply a gentle pressure with the probe and observe how the vein collapses slightly (34) (please see Video S1 in Supplementary Material). The nature of the vessels can also be confirmed by Doppler examination of the vessels. In neonates, the diameter of the intrathoracic SCV often diminishes considerably during spontaneous respiration, which renders the puncture more difficult and increases the risk of accidental pneumothorax. This phenomenon is less pronounced in children that are being ventilated by positive intermittent pressure. Although it is not impossible to successfully puncture the SCV even in very small neonates who are breathing spontaneously (please see section on sedation above), in our experience, this maneuver should only be performed by experience personnel. In the newborn, and particularly in very low weight, the radius of the J-shaped curvature of the metallic wire guide is sometimes equal to or greater than that of the vein. This can cause difficulties in its introduction. For this reason, nitinol (nickel–titanium) super-elastic shape memory guide wire is preferred because of its plicature resistance. It is recommended not to use the needle for the puncture of the vessel in very low weight neonates, but rather to use the short IV catheter supplied with the central catheter or IV catheter (24 G). Once the spontaneous or sucked reflux of blood has been obtained, the short IV catheter is introduced without the needle to the custody. This increases the chances of success and prevents the needle from moving (33). This technique is used in our service in newborns of very low weight (<1,500 g).

## Possible Complications

Central venous catheters are associated with several known complications, including mechanical complication at the time of catheter placement (accidental arterial puncture, arteriovenous fistula, pneumo- and hemothorax), catheter-related infection, thrombosis, and other complication (arrhythmias, and infiltration into adjacent tissues including pericardial and pleural spaces) (35). As centrally placed CVCs have a much lower complication rate than inadequately placed peripheral CVCs, adequate line placement confirmation is needed to minimize the complication risks (36). A too shallow position in the SVC as opposed to the SVC–RA junction has been linked to an increased risk of deep thrombosis (37). However, a deeper position of the central catheter in the right atrium has been linked to serious complications such as pericardial effusion and cardiac tamponade and prevalence of pericardial effusion/cardiac tamponade has been reported to be 1–3% (38). However, a recent meta-analysis has not been able to identify an increased risk of pericardial effusion/cardiac tamponade in children with CVCs when compared to children with peripheral venous catheter (39). Still, the recommendation of avoiding the right atrium position of the catheter in the pediatric population is based on a precautionary principle (40).

## Recommended Catheter TIP Position

The optimal position of the catheter has been subject to discussion (41) but the junction of the right atrium to the superior vena cave (SVC–RA junction) is generally regarded as a safe location for the catheter tip with the catheter’s long axis parallel to the superior vena cave, and the tip of the catheter just above the atrium itself (1, 4). In adults, guidelines from the American Society of Anesthesiologists (42), the British Committee on Standards in Hematology (43), and others (44, 45) recommend a tip position in the lower third of the SVC or the junction between the SVC–RA junction whereas other guidelines from scientific societies such as the National Kidney Foundation Kidney Disease Outcome Quality Initiative (NKF KDOQI) and the European Society for Clinical Nutrition and Metabolism accept a position in the right atrium (46, 47). In children, guidelines from the Pediatric Special Interest Group of Association for Vascular Access recommends a position in the SVC or at the SCV–RA junction (48). In our unit, we aim for a position of the catheter tip in the SVC just outside the right atrium.

## Use of US to Determine Catheter Placement

Several methods have been suggested for determining the adequate length for CVC insertion including plain radiographs and other variants of radiography, intra-atrial electrocardiographs monitoring, fluoroscopic guidance, and US (49). Most clinicians employ radiologic imaging to confirm the position (50), yet, no method is regarded as gold standard (13). Importantly, real-time US has also been demonstrated to be useful in determining the optimal catheter position and furthermore, US imaging is also useful as a screening test to diagnose catheter malposition and procedure-related complications such as thrombosis or pneumothorax (51).

It has been proposed to use a standard portable US device equipped with 3- and 6-MHz phased array transducers and a 12-MHz linear array transducer (52). First, a visualizing of the catheter by the long- and short-axis planes from a subcostal view should be attempted (52). If the catheter tip cannot be identified from the subcostal view, a right parasternal view and suprasternal coronal views of the SVC should be obtained and finally, a scan of the jugular and subclavian-brachiocephalic veins with the 12-Hz linear transducer can be attempted (52). Based on our experience, we suggest real-time verification of catheter position before fixation using the above described technique (Figure 7). All catheter positions are subsequently controlled by standard X-ray, although this was recently challenged in older children (53).

## Conclusion

In recent times, several studies on US-guided SCV cannulation in children and neonates have demonstrated not only a high overall success rate but also that the technique has relatively few complications. In our experience, the abovementioned technique offers a safe and practical alternative to cannulation of IJV and FV in children and neonates, where the use of the UV and/or PICCs is not an option. Importantly, this technique has been reported to be feasible even in children weighing less than 1,000 g. Our experience is in consistence with the literature, as we find this technique to be both feasibly and relatively free of complication when applied to the correct patients. The exact role of US-guided central catheters vs. PICCs is still to be defined. Although most neonates will not require CVCs, we believe this is the vascular access of choice in critically ill neonates and premature infants.

## Author Contributions

Drafting and revision of the work and final approval of the submitted publication: all authors.

## Conflict of Interest Statement

The authors declare that the research was conducted in the absence of any commercial or financial relationships that could be construed as a potential conflict of interest.

## Supplementary Material

Video S1 Video demonstrating the in real-time, the ultrasound (US)-guided cannulation of the brachiocephalic vein. The video starts with a short axis view of the vessels. Notice how internal jugular vein is identified by observing how it collapses with compression (between 2 and 5 s). The internal jugular vein is then followed down to its confluence with the brachiocephalic vein (comes into view at 6–7 s). Subsequently, the view is changed to the long axis view, demonstrating the brachiocephalic vein (at 8–10 s). The patient is the then punctured, and the advancement of the needle can be seen from 13 s onward. Once the needle is seen securely entering the vein (at 21–23 s) and blood can be aspirated in the syringe (not shown on the video), the US probe is removed and the guide wire is advanced. See text for details.
